# AAV for Gene Therapy in Ocular Diseases: Progress and Prospects

**DOI:** 10.34133/research.0291

**Published:** 2023-12-22

**Authors:** Xiaoyu He, Yidian Fu, Liang Ma, Yizheng Yao, Shengfang Ge, Zhi Yang, Xianqun Fan

**Affiliations:** ^1^ Department of Ophthalmology, Ninth People’s Hospital, Shanghai JiaoTong University School of Medicine, Shanghai, China.; ^2^ Shanghai Key Laboratory of Orbital Diseases and Ocular Oncology, Shanghai, China.; ^3^ Jiangsu Key Laboratory of Neuropsychiatric Diseases and Institute of Neuroscience, Soochow University; Clinical Research Center of Neurological Disease, The Second Affiliated Hospital of Soochow University, Suzhou, China.

## Abstract

Owing to the promising therapeutic effect and one-time treatment advantage, gene therapy may completely change the management of eye diseases, especially retinal diseases. Adeno-associated virus (AAV) is considered one of the most promising viral gene delivery tools because it can infect various types of tissues and is considered as a relatively safe gene delivery vector. The eye is one of the most popular organs for gene therapy, since its limited volume is suitable for small doses of AAV stably transduction. Recently, an increasing number of clinical trials of AAV-mediated gene therapy are underway. This review summarizes the biological functions of AAV and its application in the treatment of various ocular diseases, as well as the characteristics of different AAV delivery routes in clinical applications. Here, the latest research progresses in AAV-mediated gene editing and silencing strategies to modify that the genetic ocular diseases are systematically outlined, especially by base editing and prime editing. We discuss the progress of AAV in ocular optogenetic therapy. We also summarize the application of AAV-mediated gene therapy in animal models and the difficulties in its clinical transformation.

## Introduction

Hereditary eye diseases are a group of disorders attributed to genetic defects. There are approximately 200 types of hereditary ocular diseases and over 400 types of systemic diseases with ocular manifestations [1]. Single-gene inherited ocular diseases can be divided into autosomal dominant inherited diseases, autosomal recessive inherited diseases, X-linked inherited diseases, dual gene inherited diseases, and mitochondrial inherited diseases according to their genetic patterns [2,3]. Management of most types of hereditary ocular diseases is symptomatic. The loss of visual function caused by hereditary eye diseases is often difficult to cure through traditional surgery, laser therapy, and local medication. Correcting genetic defects through various methods has become the most important means of treating such diseases [4].

Gene therapy, which is extensively regarded as the utilization of exogenous DNA to treat hereditary diseases, was put forward as a potential treatment options decades ago [5,6]. Therapeutic material can be transferred to certain cells by chemical or physical delivery method to treat diseases by correcting defective genes [7,8]. Gene therapy could achieve a long-lasting therapeutic effect by using in vivo or ex vivo strategies. Genetic material was directly transferred into the target tissue or organ to work for in vivo gene therapy. While for ex vivo gene therapy, target cells were collected from patients, genetically modified, and reinjected into the patient’s body [9,10]. Gene therapy in various types has brought clinical benefits to patients with congenital blindness, spinal muscular atrophy, and hemophilia B [11]. An increasing variety of gene delivery strategies have been developed for the treatment of ocular diseases, and several substantial progresses have been achieved in this field. The benefits of gene therapy are obtained by replacing mutant genes with healthy copies or correcting potential mutations before cell degeneration. For some ocular diseases, early medical intervention can improve the survival rate of photoreceptors, thus leading to life-long benefits.

Lentiviral vectors, adenovirus vectors, adeno-associated virus (AAV) vectors, and retroviral vectors are efficient viral vectors that are widely applied in clinical and preclinical studies [12]. Retroviral and lentiviral vectors seemed to be an attractive choice for ex vivo gene therapy, while the effectiveness and safety of AAV vectors in clinical applications of in vivo gene therapy have been well confirmed [11,13]. In the past few decades, there has been a great progress toward realizing the potential of AAV-vector-mediated gene delivery for ocular disorders. Compared with other delivery systems, AAV has some advantages (transducing different types of human cells, nonpathogenic, replication-deficient, rare host cell genome integration and mediating long-term gene expression from vectors in postmitotic or slowly proliferating cells) and is considered to be one of the standard gene delivery methods for gene therapy of ocular disorders [14–17]. This success was based on many studies initiated by several groups, one of which continued to turn it into a commercial product [18–20].

**Fig. 1.**
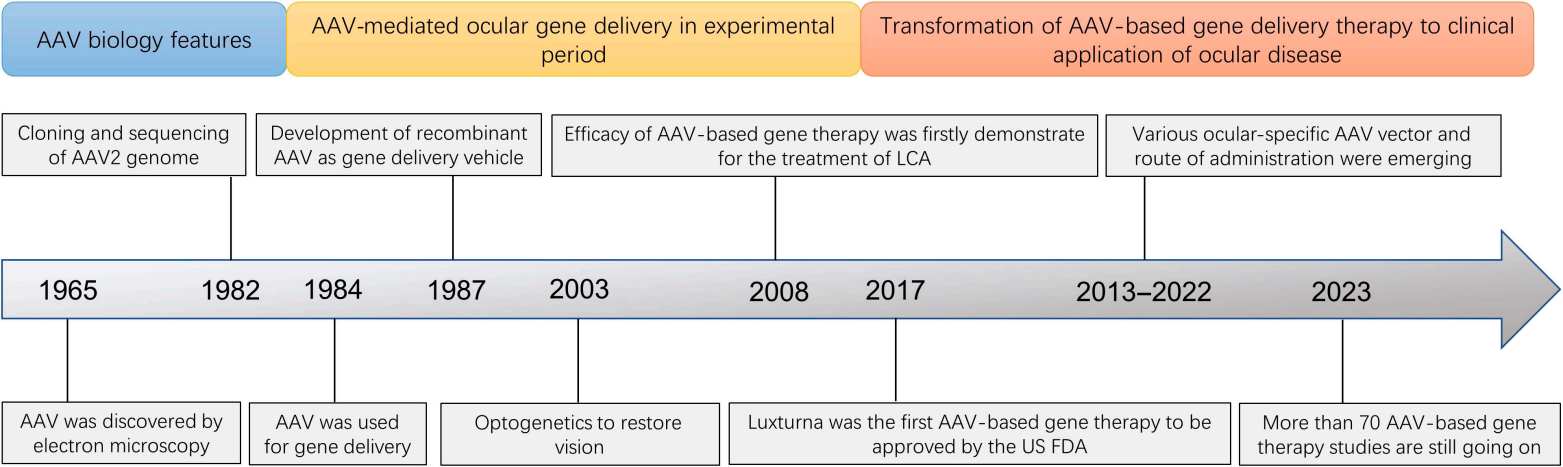
The development history of AAV as a delivery tool for ocular gene therapy. The blue box represents the stage where humans have gradually explored the biological characteristics of AAV through cloning and sequencing since its discovery. The yellow box represents the stage in which humans attempt to stably express genes in animals and humans by continuously optimizing rAAV, and, for the first time, the effectiveness of AAV-based gene therapy has been verified in LCA patients, demonstrating the enormous potential of AAV-based gene therapy in the application of ocular diseases. The red box represents the stage of AAV-based gene therapy in the bench-to-bed stage of ocular diseases. At this stage, the efficacy and safety of AAV-based gene therapy have been further improved by screening promoters and AAV capsids specifically expressed in eye tissue and developing various viral delivery pathways. The increasing number of clinical trials targeting various eye diseases has also accumulated data for the application of AAV-based gene therapy in eye diseases.

AAV was first discovered using electron microscopy during the adenovirus preparation process and subsequently isolated from human tissues [21,22]. With the cloning of wild-type (WT) AAV2 sequences, more biological characteristics of AAV have been elucidated [23–25]. Soon after AAV was vectorized for gene delivery, the development of a trans completion system made it possible to produce recombinant AAV (rAAV) with high quality, promoting the application of AAV in vivo gene therapy [26–28]. In 1995, AAV was first used in a human patient to treat cystic fibrosis [28], and then the therapeutic effect of AAV-based gene therapy was verified in the treatment of Leber congenital amaurosis (LCA) patients [18–20]. Ideal results from the first ophthalmic randomized controlled trial in gene therapy prompt the approval of Luxturna, an AAV2-based medication for LCA associated with retinal pigment epithelial 65 (RPE65), by the Food and Drug Administration (FDA) in 2017 [29]. Since then, gene therapy has gained its place in the treatment of eye diseases, and several clinical trials are underway to tackle other ocular monogenic disorders using this strategy (Fig. 1). Degenerative diseases of the retina can occur at any stage of life and mostly lead to severe visual loss [1]. Retinal degenerations address enormous and heterogeneous types of disorders, with genetic abnormalities and predisposing factors determining the disease pathogenesis [30]. Some of them, such as retinitis pigmentosa (RP) and other types of inherited retinal degenerations (IRDs), carry mutations in one or more genes, which are expressed in the RPE or photoreceptor cells of the retina, leading to severe visual impairment or even total loss of light perception [31]. These mutations may deactivate certain genes that are associated with biosynthesis, phototransduction, and folding of the rhodopsin (RHO) protein [32]. RP is a typical IRD that frequently carries mutations in RHO gene. Within RP, the most common mutation found in RHO gene disturbs the biological activity of RHO protein, leading to cellular dysfunction of photoreceptors [33]. Another group of degenerative disorders affecting photoreceptor cell survival is a complex disorder with various genetic and environmental factors. Age-related macular degeneration (AMD) is an ocular disorder with complex onset. A series of genetic, proteomic, environmental, and cellular changes were proven to be risk factors for AMD, and various therapeutic strategies are required to treat these complex retinal degenerations [34]. Small molecular compounds have been identified as potential treatments for ocular diseases. Ciliary neurotrophic factor, a member of the interleukin-6 family of cytokines, could decrease photoreceptor loss during retinal degeneration [35]. Intraocular injection of purified recombination ciliary neurotrophic factor has been demonstrated to rescue photoreceptors in different types of RP animal models, while a preclinical study showed that ciliary neurotrophic factor treatment leads to a dose-dependent increase in retinal thickness in RP patients [36]. A category of gene-coding proteins, including vascular endothelial growth factor (VEGF), tissue inhibitor of metalloproteinase-3 (TIMP-3), and Fibulin 5, was considered to be risk factors for AMD. Of these, VEGF plays a dominant role in directly and indirectly influencing vascular neovascularization and the progression of AMD. VEGF is currently regarded as the target of anti-AMD therapies [34]. Anti-VEGF antibody therapies such as ranibizumab and bevacizumab have led to current best practice guidelines in treating AMD [37]. However, overall, small molecular treatments for ocular diseases have marked limitations that make them less useful. The limited therapeutic effects of traditional medicine were possibly due to a lack of specificity and the demand of periodic administration, and a genetic-defect-targeting treatment strategy for each type of ocular diseases may be more promising [38]. Massive mutations in inherited ocular diseases, especially IRDs, have been identified, and with continuous understanding of the pathogenesis of the disease, new treatments are emerging.

Until 2023, more than 70 AAV-mediated clinical trials of ocular gene therapy have been started, most of which are in phase I or phase II. Examples include achromatopsia (AAV-CNGA3; NCT03758404 and NCT03001310), X-linked RP (XLRP) [AAV8-RP guanosine triphosphatase regulator (RPGR); NCT03116113], choroideremia (CHM) [AAV2-Rab escort protein-1 (REP1); NCT03507686], and neovascular AMD (rAAV-sFlt-1; NCT01494805) [31]. Some of these ocular disorders are caused by monogenic mutations, while the pathogenesis of others is more complicated. Nevertheless, these preclinical researches have not been consistently transferred to drugs for clinical application as reliably as the results observed by Luxturna in the treatment of LCA2. The reason for this diversity is yet to be fully elucidated, possibly because of the inefficiencies of the promoters and regulatory elements of AAV vector in target cells, and, at the same time, immune response may also be involved [39–41]. Typically, AAV vectors offer a 4.7-kb carrying capacity [42,43]. However, there are a lot of disease-causing genes that exceed AAV’s carrying capacity. One approach to extending the packaging capacity of AAV relies on the invention of a dual vector system in which transgene expression cassettes (>4.7 kb) are split into 2, and each part is packaged within an individual vector. After both vectors were transfected into the same target cell, inverted terminal repeat (ITR)-mediated recombination may result in full-length transgene expression [44,45]. Together, these developments have made AAV-mediated gene therapy for treating ocular diseases more accessible.

In this review, we mainly discussed AAV-mediated gene therapy for ocular diseases, which, to date, represents the majority of ocular gene therapy programs, despite that other viral vector platforms (lentivirus, virus-like particle) have been tested in ophthalmic clinical studies. We have summarized recent advances in AAV-mediated gene therapy for treating ocular diseases, particularly the main characteristics and delivery strategies of AAV that should be optimized to guarantee therapeutic effect, including the choice of AAV vectors and injection routes, and vector genome design. In addition, we discuss the gene editing and gene silencing strategies, along with recent research progress in various ocular diseases and their potential targets.

## Biological Features of AAV

**Fig. 2.**
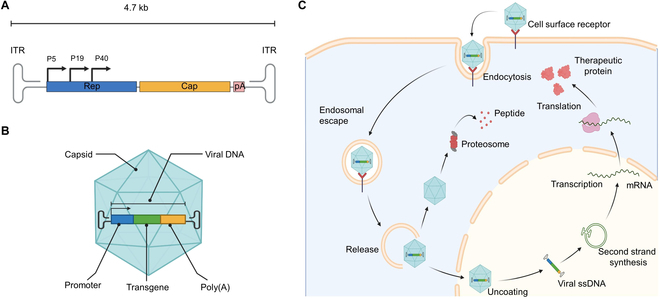
AAV biology and steps of AAV transduction in cells. (A) The blue boxes represent the Rep gene of WT AAV. The yellow box represents the viral capsid gene. The pink boxes represent assembly-activating protein (AAP) and membrane-associated accessory protein (MAAP) genes that activate and assist virion assembly. All these open reading frames are flanked by ITRs, which form T-shaped hairpin ends. (B) The Rep and Cap in rAAV were removed and replaced with a transgene expression cassette flanked by ITRs. (C) Step 1: The protein capsid of the AAV virion binds to receptors or coreceptors on the cell surface. Step 2: Activation of the endocytosis signaling pathway leads to the formation of endosomes. Step 3: Some endosomes containing AAV are ubiquitinated and labeled by proteasomes, which mediate degradation, while the other part is intracellularly trafficked to the nucleus of target cells, uncoating and releasing AAV genome DNA. Step 4: The AAV single-stranded DNA (ssDNA) genome transforms into double-stranded DNA in the nucleus. scAAV typically does not have this step, so it has a shorter transduction period. Step 5: AAV DNA is transcribed into mRNA, followed by nuclear export. Step 6: mRNA undergoes translation and posttranslational modification to form therapeutic proteins. Created with BioRender.com. Poly(A), polyadenylate.

WT AAV is a kind of nonenveloped parvovirus with single-stranded DNA, the genome of which comprises Rep and Cap gene flanked by 2 ITRs (Fig. 2A) [46]. AAV is a dependovirus, needing the assistance of helper virus for DNA replication with a particle size of 24 to 26 nm in diameter [47]. Researchers found that although AAV itself exists in people infected with adenovirus or herpesvirus and other auxiliary viruses, it does not cause any disease [46]. The Rep gene encodes 4 key nonstructural proteins (Rep40, Rep52, Rep68, and Rep78) that are crucial for genome integration, replication, transcriptional regulation, and virion assembly. With the assistance of the assembly-activating protein, Cap gene encodes 3 structural proteins (VP1, VP2, and VP3) that constitute a 60-mer viral capsid [48,49]. The viral genome is packed into an icosahedral capsid composed of 60 VP subunits, which are assembled in a 1:1:10 stoichiometry of VP1:VP2:VP3. Each subunit contains 9 variable regions (VRs) on the surface of AAV virions, which decide the natural tropism and intracellular transport of AAV and are generally the domains for neutralizing antibody recognition [50,51]. These VRs make a difference between naturally occurring AAV serotypes identified from a wide range of vertebrates. Genetic modification of these regions can improve the AAV tropism to target cells [13,52,53]. rAAV results in packaging transgenes for therapeutic applications by dislodging all viral genomic components except for ITRs. In trans form, Rep and Cap are provided in the production process of AAV virions (Fig. 2B). With the progress of rAAV technology, the helper genes required for AAV replication have been clarified and may be cloned into plasmids for virion production. At present, the 3-plasmid cotransfection system developed more than 20 years ago still occupies the mainstream position of AAV gene therapy. The 3 plasmids include adenovirus helper plasmids (E4, E2a, and VA genes), plasmids with gene of interest, and packaging plasmids with the Cap and Rep genes. Three plasmids need to be transfected into the host cell (human embryonic kidney 293 cells in most cases) to assemble rAAV virus particles.

To date, at least 13 natural AAV serotypes have been identified, and more than 100 variants have been isolated and investigated as gene delivery vehicles. Among these vectors, AAV mutants are still being generated and detected, expanding the application of AAV for gene delivery [52]. Some serotypes, including AAV1, AAV2, AAV5, AAV6, AAV8, AAV9, and AAVrh10, and their associated mutants, like AAV2.5, AAV2tYF, and AAV.7m8, have been applied in clinical trials for various types of diseases [54–56]. It has been confirmed that the diverse expression of AAV receptors in different cell types and tissues affects the tissue propensity of AAV [57]. For example, the major cell surface receptor of AAV2 is heparin sulfate proteoglycan, while the laminin receptor initiates AAV8 attachment to the host cell [46,58]. AAVR, also known as KIAA0319L, is a newly identified multiserotype AAV receptor that is essential for AAV transduction and is found in a variety of organs, including the liver, lung, kidney, and retina [59,60]. AAV2, AAV5, and AAV8 are the most commonly used serotypes in the field of ocular disease treatment. AAV2 has a natural preference for RPE cells, which makes it an ideal choice for AAV-mediated gene therapy of RPE-related IRDs. In addition, AAV5 and AAV8 are more representative for efficient transduction of photoreceptors and are therefore widely applied in clinical trials to target photoreceptors (rods and cones) [61]. The transduction process of the AAV vector includes several steps. AAV infects target cells by attaching to the cell surface under the joint action of primary receptors and coreceptors, triggering endocytic signaling in cells. Next, AAV capsid exposes the N terminus of the vp1-2 protein, and virions are then released from endosomes and accumulate around the perinuclear region. AAV virions uncoat and release their single-stranded DNA and then convert it into double-stranded DNA after nuclear import. The transgene could complete a series of procedures, such as transcription, translation, and posttranslational modification, and finally form a therapeutic protein in the target cell [9,46,47]. This enables the transgene to be permanently expressed in nondividing cells, making postmitotic tissues, such as the retina, an ideal target for AAV-mediated gene therapy (Fig. 2C). The viral DNA primarily remains as extrachromosomal form, and the integration frequency of AAV genome is decreased to about 0.1% [62]. Mutations that are deleterious to the host are also less likely to occur as a result of the lower integration frequency, which therefore improves the safety of clinical application [62].

## AAV-Vector-Based Gene Replacement for Ocular Disease

Early research on the biological characteristics of AAV laid a foundation for its application in mammalian gene therapy [25,63,64]. Today, rAAV is regarding as the ideal platform for in vivo gene delivery, and 5 AAV-based gene delivery medication [approved by FDA/European Medicines Agency (EMA)] are in clinical application (Table 1). To date, researchers are still conducting in-depth research to improve the safety and effectiveness of AAV-based gene therapy.

**Table 1.** AAV-based gene therapy products

Name	Sponsor	Condition	Approval time	Regulatory agency
Glybera	uniQure	Lipoprotein lipase deficiency	2012 (withdrawal in 2017)	EMA
Luxturna	Sparke Therapeutics	LCA	2017	FDA
Zolgensma	Norvartis	Spinal muscular atrophy	2019	FDA
Upstaza	PTC Therapeutics	Aromatic l-amino acid decarboxylase deficiency	2022	EMA
Roctavian	BioMarin Phamaceutical	Hemophilia A	2022	EMA/FDA
EtranaDez	uniQure/CSL Behring	Hemophilia B	2022	FDA

**Fig. 3.**
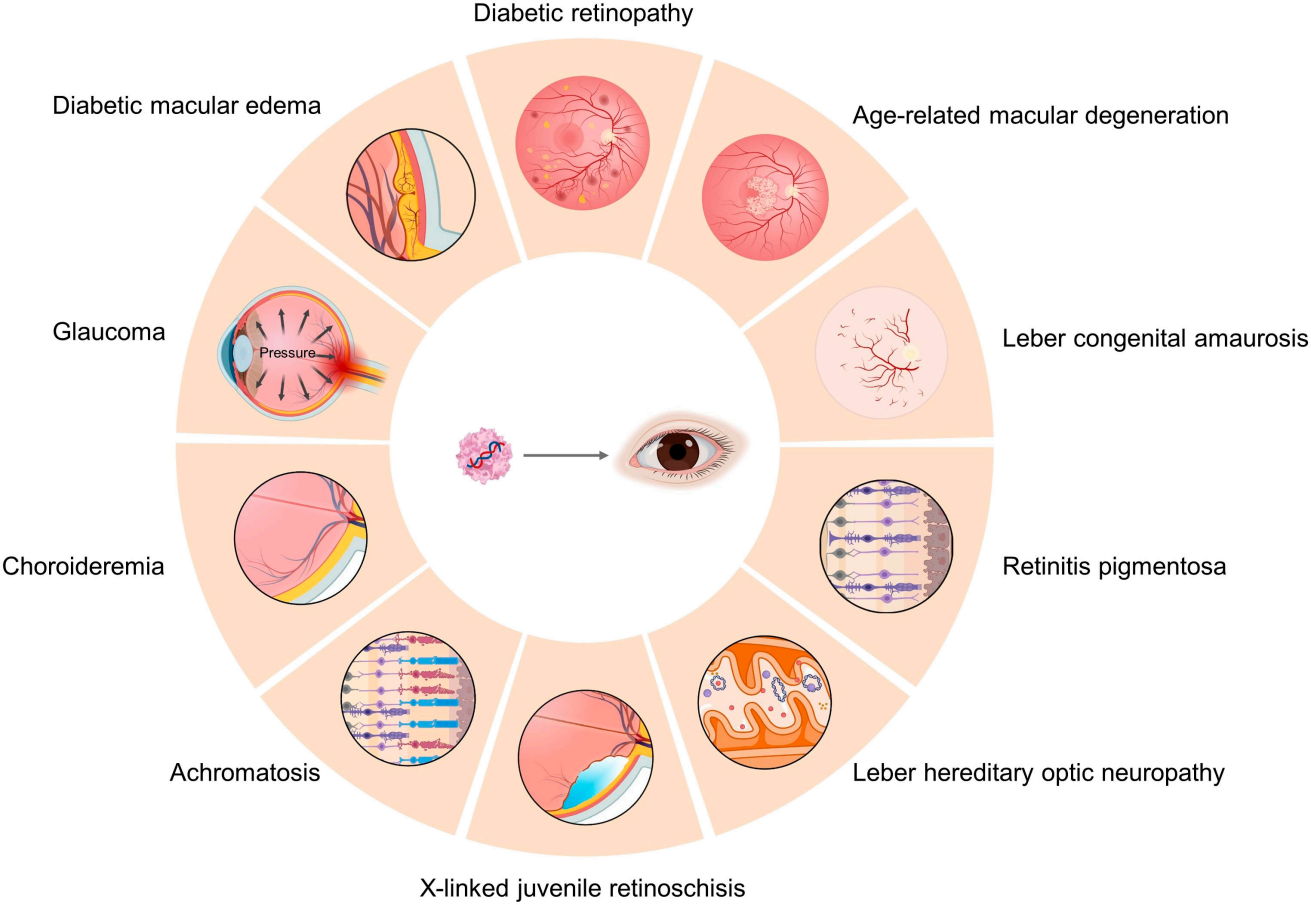
AAV-mediated gene therapy for various types of eye diseases. Schematic diagram of AAV-mediated gene therapy for different eye diseases. DME: Yellow protrusions on the retina represent macular edema. DR: Fundus photos show vascularization, bleeding, and exudation. AMD: The fundus photo shows drusen (yellow dot) in the macular area. LCA: Fundus photos show narrowing of small artery blood vessels, whitening of the optic disc, and macular atrophy. RP: Damage, atrophy, or loss of photoreceptor cells (cone cells and rod cells). LHON: Mitochondrial genetic disease in which RGCs and their axons undergo degeneration. XLRS: A typical manifestation of retinal splitting. Acromatosis: Complete cone cell dysfunction. Choroidemia: Complete absence of the choroidal layer between the sclera (gray) and the retina (yellow). Glaucoma: A common eye disease caused by increased intraocular pressure. Created with BioRender.com.

AAV is one of the most promising gene delivery tools for treating ocular disease. To date, many AAV-based gene therapies are in clinical trials, which are listed in Table 2. Currently, strategies of most clinical trials of ocular gene therapy are to deliver WT copies of transgenes to photoreceptor or RPE cells. In general, basic AAV gene delivery vector can consist of 2 ITRs, promoters, cDNA of transgene, enhancers, or other transcriptional stabilizing elements and polyadenylate tail sequences [65]. Immunity privileges in a relatively closed environment and the controllable nature of the treatment process have made gene therapy for ocular diseases, especially retinal diseases, a popular topic in the past 20 years [66]. Here, we summarize clinical studies on ocular diseases based on AAV-vector-mediated gene therapy (Fig. 3).

**Table 2.** Completed and ongoing gene replacement trials for ocular disease

Disease	Target	Delivery	Vector	Phase	Sponsor	NCT number
LCA	RPE65	SR	AAV2	I/II	Spark Therapeutics	01208389
	RPE65	SR	AAV2	I/II	Spark Therapeutics	00516477
	RPE65	SR	AAV2	III	Spark Therapeutics	00999609
	RPE65	SR	AAV2	I/II	UCL	00643747
	RPE65	SR	AAV5	I/II	MeiraGTx UK II Ltd	02781480
	RPE65	SR	AAV6	I/II	MeiraGTx UK II Ltd	02946879
	RPE65	SR	AAV2	I/	Hadassah	00821340
	RPE65	SR	AAV2	I/II	AGTC	00749957
	RPE65	SR	AAV2	I	UPenn	00481546
	RPE65	SR	AAV2/4	I/II	Nantes	01496040
RP	PDE6A	SR	rAAV	I/II	STZ eye trial	04611503
	PDE6B	SR	AAV2/5	I/II	Horama S.A.	03328130
	RLBP1	SR	AAV8	I/II	Novartis Pharmaceuticals	03374657
	NR2E3	SR	AAV5	I/II	Ocugen	03326336
	MCO	IVT	AAV2	I/II	Nanoscope Therapeutics Inc.	04919473
	MERTK	SR	AAV2	I	Fowzan Alkuraya	01482195
LHON	ND4	IVT	AAV2	I	Huazhong	02161380
	ND4	IVT	AAV2	I/II	Huazhong	01267422
	ND4	IVT	AAV2	II/III	Huazhong	03153293
	ND4	IVT	AAV2	I/II	GenSight Biologics	02064569
	ND4	IVT	AAV2	III	GenSight Biologics	02652780
	ND4	IVT	AAV2	III	GenSight Biologics	02652767
	ND4	IVT	AAV2	III	GenSight Biologics	03293524
	CNGA3	SR	AAV2	I/II	Applied Genetic Technologies Corp	02935517
XLRS	RS1	IVT	AAV8	I/II	NEI	02317887
	RS1	IVT	AAV2tYF	I/III	AGTC	02416622
ACHM	CNGA3	SR	AAV2/8	I/II	MeiraGTx UK II Ltd	03758404
	CNGA3	SR	rAAV	I/II	STZ eyetrial	02610582
	CNGB3	SR	AAV2/8	I/II	MeiraGTx UK II Ltd	03001310
	CNGB3	SR	AAVtYF	I/II	AGTC	02599922
CHM	REP1	SR	AAV2	I/II	Ian M. MacDonald	02077361
	REP1	SR	AAV2	I/II	University of Oxford	01461213
	REP1	SR	AAV2-hCHM	I/II	Spark Therapeutics	02341807
	REP1	SR	AAV2	II	STZ eyetrial	02671539
	REP1	SR	AAV2	II	Byron Lam	02553135
	REP1	SR	AAV2	II	UCL	02407678
	REP1	SR	AAV2	II	NightstarRx Ltd	03507686
	REP1	SR	AAV2	III	NightstarRx Ltd	03496012
	REP1	SR	R100	I	4D Molecular Therapeutics	04483440
XLRP	RPGR	SR	AAV2/5	I/II	MeiraGTx UK II Ltd	03252847
	RPGR	SR	AAV2tYF	I/II	AGTC	03316560
	RPGR	SR	AAV8	I/II	NightstarRx Ltd	03116113
	RPGR	IVT	R100	I/II	4D Molecular Therapeutics	04517149
DR	Anti-VEGF	SC	AAV8	II	Regenxbio Inc.	04567550
DME	Aflibercept	IVT	AAV7	II	Adverum Biotechnologies	04418427
AMD	Aflibercept and anti-VEGF-C miRNA	IVT	R100	II	4D Molecular Therapeutics	05197270
	Anti-VEGF	SC	AAV8	II/III	Regenxbio Inc.	04514653
	Anti-VEGF	SR	AAV8	II	Regenxbio Inc.	04832724
	Anti-VEGF	IVT	AAV8	II/III	Regenxbio Inc.	04704921
	Aflibercept	IVT	AAV7	I	Adverum Biotechnologies	03748784
	CFI	SR	AAV2	II	Gyroscope Therapeutics Limited	03846193
	sFLT-1	IVT	AAV2	I	Genzyme/Sanofi	01024998

sFLT-1, soluble FMS-like tyrosine kinase 1; CFI, complement factor I; SC, suprachoroidal injections; MCO, multicharacteristic opsin; NR2E3, nuclear receptor subfamily 2, group E, member 3; CNGA3, cyclic nucleotide-gated channel α-3; CNGB3, cyclic nucleotide-gated channel β-3.

## Leber congenital amaurosis

LCA is a hereditary retinal disease characterized clinically by severe early vision loss, sensory nystagmus, melanoma, and lack of response to electroretinogram (ERG) signaling [67]. RPE65, which encodes the isomerase essential for the retinoid visual cycle, converts all trans retinol esters to 11-cis-retinol. Three to 16% of LCA patients carry biallelic mutations in RPE65 gene, and RPE65-associated LCA is also one of the earliest diseases explored for gene therapy [68].

Currently, there are more than 10 clinical trials exploring the effectiveness and safety of a subretinal (SR) injection of AAV2 vectors carrying RPE65 cDNA for LCA cases with biallelic RPE65 mutations. In a phase III randomized controlled trial (RCT, NCT00999609), 31 participants diagnosed with RPE65-associated inherited retinal dystrophy (21 interventions:10 controls) were sequentially treated with AAV2-hRPE65v2. The effectiveness of this trial was determined by the change in the multiluminance mobility test score over 1 year. Considering the growth of the retina, patients younger than 12 months were excluded. The results showed that after 1 year of treatment, the average bilateral multiluminance mobility test change score of the intervention group was 1.8, while the control group was 0.2 (*P* = 0.0013). After confirming the efficacy of Luxturna, the study also discussed the safety issues that may be caused by it, including high intraocular pressure (20%), cataracts (15%), inflammation (10%), retinal tears (10%), and eye pain (5%) [69]. Recently, Atsena Therapeutics released its data on an AAV5-based gene therapy for guanylate cyclase 2D (GUCY2D)-associated LCA1 (NCT03920007). The results showed clinically substantial improvements in retinal sensitivity, best corrected visual acuity (BCVA), and other visual functions in subjects receiving the highest dose, and there were no drug-related adverse events.

However, it has some potential defects that need to be considered. A recent review collected data from 6 studies (5 prospective nonrandomized clinical trials and 1 randomized) and demonstrated that the efficacy of Luxturna seems to be limited to within 2 years after treatment. In addition, patients treated with it had thinner central retinal thickness [70]. It still has the potential to further improve and prolong the effective time of it. Although Luxturna is a pioneering therapeutic drug in human gene therapy, it is far from being a perfect gene therapy drug; it still has a long way to go.

## Retinitis pigmentosa

RP is a widespread category of IRDs. In RP, mutations usually occur in the RHO gene, influencing the biological function of RHO and indirectly affecting the function of rods [33]. RP patients usually lose night vision at an early age and side vision in their adulthood and gradually lose central vision with the loss of photoreceptors later in life [71]. One mechanism of RP is that these mutations cause the truncation of RHO, which affects the ability of cells to fold and transport proteins, thus resulting in the imbalance of cellular homeostasis. Another mechanism suggests that RP is caused by the abnormal expression of RHO [72]. More than 60 causative genes associated with RP with various types of pathogenic mutations have been identified. MER proto-oncogene, tyrosine kinase (MERTK), phosphodiesterase 6B (PDE6B), and retinaldehyde binding protein 1 (RLBP1) are the 3 most common targets for gene therapy, and 3 clinical trials corresponding to these genes are underway. MERTK has been reported in a phase I clinical trial to become a therapeutic target, with 6 patients receiving SR rAAV2-vitelliform macular dystrophy 2 (VMD2)-hMERTK treatment containing human MERTK genes driven by RPE-specific VMD2 promoters and followed up for 2 years. The results showed that there were no related complications, and 50% of patients showed improvement in vision within the first month after treatment. However, 2 of 3 patients returned to their baseline vision level 2 years later [71]. These 2 patients developed cataracts, but it is not yet clear whether it is related to vitrectomy or RP progression.

Nonsyndromic RP refers to only retinal malnutrition without any other organs being affected and can be classified into 3 categories: autosomal recessive, autosomal dominant, and X-linked [73]. Since the genetic pattern of the disease, there are 2 main gene therapy methods available for RP. For recessive RP, the loss function of target protein can be treated through gene supplementation methods. In dominant RP, gene therapy methods include gene silencing with or without gene complementation [74].

### Leber hereditary optic neuropathy

Leber hereditary optic neuropathy (LHON) is a maternal genetic disease caused by mutations in mitochondrial DNA and is common in young people. Its characteristic is acute painless central visual loss caused by retinal ganglion cell (RGC) layer and optic nerve degeneration [75]. It was demonstrated that the delivery of NADH dehydrogenase subunit 1 (ND1) and NADH dehydrogenase subunit 4 (ND4) genes by AAV into fibroblasts for transcript expression restored the activity of electron transport chain and that complementation of the defective gene by intravitreal (IVT) injection of AAV rescued vision [76,77]. The first gene therapy clinical trial for LHON evaluated the safety, efficacy, and sustainability of rAAV2-ND4 IVT injections in 9 patients with ND4 gene mutations (NCT01267422) [78]. Seven years of follow-up showed that rAAV2-ND4 gene therapy was safe, without associated systemic or ocular adverse events, and the treated eyes showed structural and functional improvements. Another phase II/III study with 142 participants validated the safety, durability, and efficacy of rAAV2-ND4 (NCT03153293). The first 3 months of this large-scale study focused on baseline functional features related to visual recovery [79]. Patients who are newly diagnosed or start gene therapy early can obtain a better curative effect.

## X-linked juvenile retinoschisis

X-linked juvenile retinoschisis (XLRS) is one of the main diseases causing macular degeneration in male children and is characterized by progressive macular atrophy and splitting of the neurosensory retina [80]. XLRS is a type of IRD caused by gene mutations in retinoschisin 1 (RS1), which plays an essential role in the intracellular adhesion of retinal cells. Patients often lose central vision in early childhood, which can be complicated by vitreous hemorrhage and retinal detachment as they age. Gene replacement therapy (AAV8-RS1) can restore retinal structure and function in mouse models [81].

To date, there are 2 ongoing AAV-mediated complementation trials for XLRS. Considering the fragility of the XLRS retina and the tendency for retinal detachment, IVT injection is certainly a superior delivery strategy over SR. Preclinical studies have demonstrated that the internal limiting membrane, a main transduction barrier for IVT AAV delivery, is weakened by XLRS, enhancing the delivery effect of SR injection [82,83]. A phase I/II trial (NCT02416622) recruiting 27 XLRS patients uses an AAV2 vector encoding the RS1 gene (rAAV2tYF-CB-hRS1). This study is a dose escalation study that focuses on participants who experience adverse events within 12 months after receiving IVT injection of AAV. This study also plans to evaluate BCVA, optical coherence tomography (OCT) to assess schisis cavity size, and b-wave amplitude in ERG response. Research has shown that almost all patients experienced eye adverse events during early follow-up, while changes in other indicators were uncertain.

## Achromatosis

Achromatosis (ACHM) is a congenital autosomal recessive disease of cone dysfunction. ACHM patients usually present with impaired photosensitivity and color vision and early-onset nystagmus. Mutations in the 5 phototransduction genes (PDE6C, PDE6H, GNAT2, CNGA3, and CNGB3) are implicated in ACHM and account for over 93% of cases (CNGB3 and CNGA3 account for over 70%) [84]. ACHM may be caused by mutations in the CNGB3 and CNGA3 genes that encode the α and β subunits of the cone guanosine 3′,5′-monophosphate-gated channel [85,86].

A recent RCT research investigated the efficacy and safety of administering CNGA3 expressing AAV8 vectors (AAV8-CNGA3) to poorer eyes in ACHM patients with biallelic pathogenic CNGA3 mutations. Within 1 year after receiving the treatment, 9 ACHM patients tolerated the treatment well, all adverse reactions were mild, and no serious side effects were observed. The color perception and contrast sensitivity of the treated eyes were improved, which indicated functional recovery of the cone [87].

## Choroideremia

CHM is a kind of X-linked recessive disease caused by CHM gene mutations, mainly characterized by progressive retinal degeneration and blindness. The pathogenic gene CHM is the coding gene for REP1 and plays a crucial role in the intracellular trafficking signaling pathway. Its disruption can damage cellular homeostasis. In CHM, cone and rod cells do not degenerate until late CHM, extending the window for gene therapy [88].

In preclinical research, SR of AAV2-REP1 in WT mice, followed 5 weeks later by immunoblot analysis, confirmed the production of human REP1 in the mouse RPE, which did not result in a toxic response when overexpressed. After 6 months of AAV injection, retinal function was evaluated by electroretinography, and the results showed a dose-dependent effect of AAV2-REP1 expression in Chm^null/wt^ mice [89].

The phase I/II clinical trial was primarily designed to evaluate the effective dosage and safety. Early results in patients receiving low-dose therapy demonstrated AAV2-REP1 gene therapy to be safe and resulted in visual acuity improvement compared with untreated eyes. The long-term follow-up of the same cohort showed that the improvement in vision caused by gene therapy lasted for 3.5 years [90]. NSR-REP1 can produce REP1 in the eyes, which is an AAV2 vector containing recombinant human cDNA and is currently in phase III clinical stage. Previously, in open-label clinical trials of 32 patients receiving NSR-REP1 treatment, over 90% of patients maintained vision during a 2-year follow-up period. These clinical experimental data have promoted the development of AAV-based gene therapy medication, making it possible to cure CHM.

## X-linked retinitis pigmentosa

XLRP is the most common recessive RP and a genetic disease caused by mutations in the RPGR gene. It is characterized by the degeneration of cones and rods in early childhood, resulting in severe sight loss and visual field constriction [91].

Considering that XLRP accounts for a considerable proportion of all RP cases, genetic intervention in RPGR-associated XLRP has been vigorously promoted [92]. A recent paper reported the initial results of the first human phase I/II clinical trial for XLRP caused by RPGR gene mutations in 18 cases with up to 6 months of follow-up (NCT03116113). SR delivery of an AAV vector encoding codon-optimized human RPGR (AAV8-coRPGR) brought visual field improvements for XLRP patients without serious adverse effects. Meanwhile, an increase in the thickness of outer nuclear layer was observed on OCT in the treated eyes, suggesting that a longer follow-up may be required to further assess its safety [91].

## Age-related macular degeneration

AMD is the main cause of irreversible central vision loss in people over 65 years old. The underlying pathology of AMD is not yet clear [93]. Recent studies suggest that drusen caused by the complement cascade is one of the triggers for AMD [94]. AMD is mainly divided into 2 types: no neovascular AMD (dry AMD) and neovascular AMD (wet AMD). For dry AMD, although developments in cancer biology have elucidated the mechanisms of neovascularization and provided effective inhibitors, little is known about the underlying pathology of dry form [93]; several AAV-based pharmaceuticals are currently target key molecules, such as complement factor I in the complement cascade. AAV delivery of soluble CD59 (AAV.CAG.sCD59, HMR59) has been developed to block complement in the membrane attachment complex. Seventeen patients with advanced stage of non-neovascular AMD participate this clinical trial (NCT03144999). Subjects receive a single IVT injection of HMR59 to assess the safety and effectiveness of the treatment. For wet AMD, anti-VEGF is still the most effective choice. Anti-VEGF therapy has been shown to have substantial benefits for neovascular AMD patients by blocking the activity of VEGF [95]. RGX-314 is an AAV8-based drug that sustains the expression of a monoclonal anti-VEGF Fab, which could neutralize VEGF activity. The ongoing clinical trials of RGX-314 (NCT04514653, NCT04832724, and NCT04704921) aim to explore their effectiveness and safety in treating wet AMD through different delivery pathways (IVT injection, SR injection, and suprachoroidal injection).

## Diabetic retinopathy and diabetic macular edema

Diabetic retinopathy (DR) is one of the main causes of adult vision loss in developed countries and one of the most common complications of diabetes. The pathological characteristics of DR are morphological changes in microvessels, including loss of tight junctions between the endothelium, thickening of the basement membrane, and loss of pericytes. These changes result in vascular permeability increasing, microaneurysms, and ultimately blindness [96]. DR leads to changes in ocular microvascular hemodynamics, which leads to retinal thickening or hard exudative deposition caused by the accumulation of extracellular fluid in the center of the macula, which is called diabetic macular edema (DME). At present, the optimal treatment for DR and DME is IVT injection of anti-VEGF. However, the efficacy of anti-VEGF antibodies is hindered by the limited half-life of protein drugs, requiring frequent IVT injection treatments, which places a heavy burden on patients [97]. AAV-based anti-VEGF products aim to target VEGF through AAV gene delivery to form long-term therapeutic effects on DR and DME by mediating continuous expression of anti-VEGF molecules. Previous studies have verified the key role of anti-VEGF molecules in the treatment of DME. It leads to a better visual improvement than focal macular laser (NCT00473382 and NCT01331681). Because of the same therapeutic targets, AAV-based anti VEGF treatment medications for AMD, including RGX-314 and ADVM-022, also have guiding significance for the treatment of DME. A phase II clinical trial evaluated the safety and tolerability of one-time IVT injection of ADVM-022 for DME treatment (NCT04418427). These clinical trials are driving the application of anti-VEGF products based on AAV in different eye diseases. At the same time, a larger patient population will make gene therapy products more economical, which will help to promote the widespread application of products in the population.

## Route of Ocular AAV Delivery

**Fig. 4.**
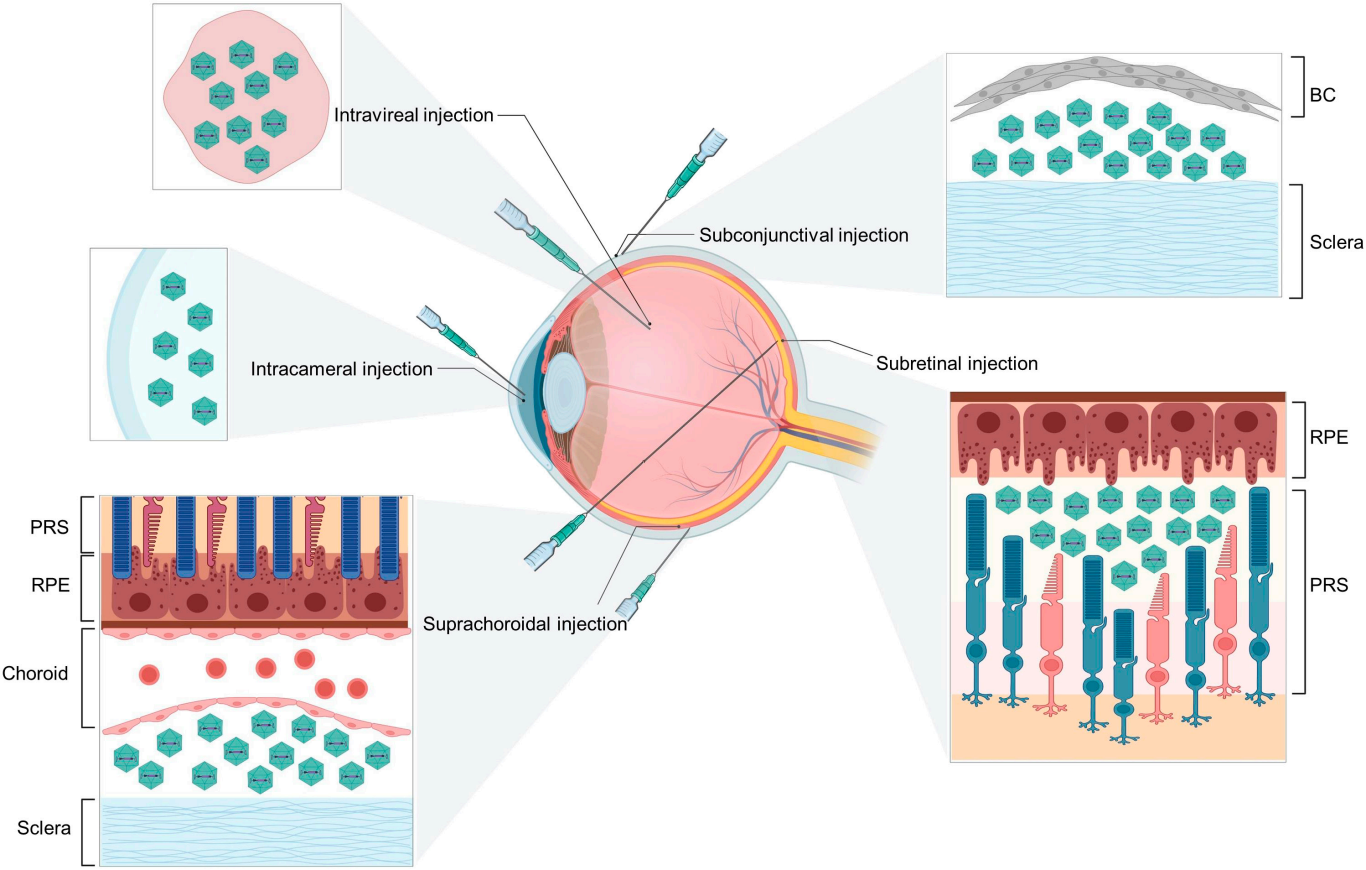
Typical AAV delivery routes for ocular gene therapy. Both IVT injection and intracameral injection leave AAV in the cavity. Subconjunctival injection preserves AAV virions in the gap between the bulbar conjunctiva (BC) and sclera. SR injection mainly leaves AAV virions in the limited gap between photoreceptors (PRS) and RPE in the retina, and suprachoroidal injection mainly leaves AAV virions in the gap between the sclera and choroid. Created with BioRender.com.

The choice of appropriate administration method depends mainly on the eye area to be medicated. Administration of the delivery route makes a large difference in the transduction efficiency and biodistribution profile of the AAV vector. Because of the limitation of blood-eye barrier, less AAV can be enriched in ocular tissues via systematic administration. Therefore, the current treatment of AAV in the eye is mainly completed by local delivery (Fig. 4) [98].

SR injection is the most well-established ocular AAV delivery route. Through SR injection, the AAV virions are transported to the SR space located between RPE cells and photoreceptors. Thus, diseases with gene defects in RPE cells and photoreceptors are more suitable for treatment with SR injection. SR injection of AAV with the same vehicle dose resulted in an increase in the copy number of transgenes in the retina and increased transduction of photoreceptors and RPE cells [99]. The AAV vector delivered by SR injection is mainly limited to the injected SR region, and the limited space under the retina makes less AAV dose required to achieve the same therapeutic effect, thus decreasing the immune response caused by the expression products or vector capsids [100]. Experienced vitreoretinal surgeons and operating rooms equipped with an operating microscope and a vitrectomy machine are crucial for SR injection surgery, which also limits its promotion. SR injection is suitable only for the treatment of IRDs or other retinal diseases in which retinal structures remain intact. Nonstandard SR injection may cause severe side effects, like vitreous hemorrhage and retinal detachment. For different types of IRDs, variations in SR injection protocols should be selected to produce the best treatment results.

Compared with SR injection, suprachoroidal injection seems to be a less invasive outpatient procedure with lower risks. According to a recent study, the transduction and therapeutic effects of suprachoroidal injection are similar to those of SR injection, which provides an alternative procedure for AAV delivery to the retina, especially to the outer retina [101,102].

In addition, IVT delivery is one of the most important routes for gene delivery to inner retinal layer, such as RGCs [103,104]. Compared with SR injection, vitreous injection is less invasive and easier to perform and can also deliver transgenes to a wider retinal area. After IVT injection, most AAV vectors cannot reach the outer retina due to the barriers of the membrane structure in the retina and the tight connections between retinal cells. Recently, some AAV variants are emerging to show the ability to penetrate through the whole layer of retina and transduce outer retinal cells in rodents [105–107]. Notably, AAV vectors delivered to the vitreous can also be transduced to the anterior segment tissues (ciliary body, corneal endothelium, and trabecular meshwork). The reason for this situation may be due to the diffusion of AAV vectors in the vitreous body into aqueous humor.

Noninvasive local administration, subconjunctival injection, and intracameral injection are the 3 most common methods of AAV anterior segment tissue delivery. Subconjunctival injection of AAV may be a simple and safe gene delivery strategy for the treatment of corneal, ocular surface, muscle, and optic nerve diseases [108]. In some instances, tissues from the posterior segment, such as the retina, were also transduced after subconjunctival administration [109]. In mice, through the strategy of intracameral injection, AAV variants can be transduced into anterior segment tissues, including corneal endothelium, trabecular meshwork, and stroma [110]. A recent study showed that by intracameral injection of AAV2 containing exozyme C3 transferase into the anterior chamber of mice and primates, genetic materials can be delivered to the trabecular meshwork, leading to a reduction in intraocular pressure [111]. By using AAV5-decorin (DCN) topically, this noninvasive gene therapy approach demonstrates the potential for treating corneal fibrosis and neovascularization in vivo without marked eye toxicity [112].

Table 3 lists more AAV variants that have been genetically delivered to eye tissues using different injection routes. Through these preclinical experiments, we can observe the delivery efficiency of AAV to different tissues of the eye through different delivery routes, considering the difficulty of various delivery routes and the invasiveness to eye tissues. Adopting reasonable delivery methods ensures the effectiveness of AAV gene therapy while enhancing the safety of treatment by avoiding unnecessary delivery. It should be noted that the animal models used in preclinical experiments are different from humans in terms of eye tissue structure [31]. For example, the mouse lens accounts for a much larger proportion than the human lens, while the inner limiting membrane of humans is also thicker than that of mice.

**Table 3.** Specific information about AAV-mediated preclinical experiments

Route of administration	Tissue	Serotype	Species	Transgene	References
Topical drops	Cornea	AAV1/2/5/7/8	Rabbit/human	GFP	[153]
	Cornea	AAV2	Rat	EGFP	[154]
	Cornea	AAV8	Mouse	Alkaline phosphatase	[155,156]
	Cornea	AAV5	Rabbit	GFP	[157,158]
	Cornea	AAV5	Rabbit	GFP/HLA-G	[159]
Intracameral	Cornea	AAV2	Rabbit	EGFP/MMP-3	[160]
	Cornea	AAV9	Mouse	GFP/LacZ	[161]
	Cornea	ssAAV2/scAAV2	Rat/NHP/mouse	GFP	[162,163]
	Trabecular meshwork cornea/trabecular meshwork/ciliary body	scAAV2/AAV8/AAV.Anc80L65	Mouse	GFP	[110]
	Cornea/trabecular meshwork/ciliary body/iris	scAAV2/scAAV5/scAAV8	Rat	GFP	[164]
Suprachoroidal	Choroid/retina	AAV8	Rat	GFP	[101]
	Choroid/retina	AAV8	NHP	GFP	[165]
Subconjunctival	Conjunctiva/cornea/muscle/optic nerve/eyelid	AAV2/6/8	Mouse	GFP	[108]
	Conjunctiva/cornea/optic nerve/eyelid	scAAV8	Mouse	GFP	[166]
	Cornea	AAV8	Mouse	EGFP/endostatin	[167]
	Conjunctiva/cornea	AAV-DJ	Rabbit	CRISPR–CTGF	[168]
SR	Choroid/retina	AAV2tYF/AAV5	Sheep	CNGA3	[169]
	Retina	AAV1/2/5/6/7/8/9	Mouse/monkey/pig	EGFP	[61,170–172]
	Retina	AAV4/5	NHP/rat/dog	GFP	[173,174]
	Retina	AAV2/AAVrh10	Mouse	RHO	[175]
	Liver/muscle/retina	AAV2/8	Pig/NHP/mouse	GFP/FIX	[176,177]
	Retina	AAV^K9#4^/ AAV^K9#12^	Dog	GFP/LRIT3	[178]
	Retina	AAV2/5	Mouse	GPF/BBS1	[179]
	Retina	AAV2	Mouse	GFP/SEMA3F	[180]
	Retina	AAV2	Human	RPE65	[181]
	Retina	AAV2	Mouse	RPGR	[182]
	Retina	AAV8BP2	Mouse	EGFP	[141]
	Retina	AAV2-Tyr/ AAV8-Tyr	Mouse	EGFP	[83]
	Retina	scAAV2	Mouse	GFP	[183]
IVT	Retina	AAV2.GL/NN	NHP/mouse/dog/human	CNGA3/EGFP	[133]
	Retina	AAV1/2/3	Dog/rat	GFP/IRBP	[184,185]
	Retina	AAV2.7m8	NHP/mouse/sheep	GFP/RPE65/CNGA3	[105,186]
	Retina	AAVrh8/ AAVrh10	Mouse	EGFP/GFAP/calbindin	[187]
	Retina/cornea/trabecular meshwork/lens epithelium/Schlemms canal/iris	AAV7/8/9	Mouse	EGFP	[170]
	Retina	AAV8BP2	Mouse	EGFP	[141]

LRIT3, immunoglobulin-like and transmembrane domain 3; BBS1, Ardet–Biedl syndrome type 1; Sema3f, semaphorin 3F; IRBP, interphotoreceptor-binding protein; GFAP, glial fibrillary accessory protein; EGFP, green fluorescent protein.

## AAV-Mediated Gene Silencing for Ocular Disorders

Compared with the replacement strategy, which is extensively used in clinical trials, rare gene silencing approaches, including ribozyme-based, interfering RNA-based, or CRISPR-based approaches, have been investigated to suppress mutant or WT genes related to the onset of ocular diseases (Table 4) [113]. The use of CRISPR-Cas is usually caused by gene knockout and deletion through the nonhomologous end joining (NHEJ) repair pathway, which is commonly used to treat genetic disease mutations with dominant or functionally acquired genes [114]. CRISPR-Cas can also achieve precise in situ repair through the homology-directed repair (HDR) pathway under the condition of endogenous templates, but the efficiency of HDR in vivo is usually low [115]. In some preclinical animal experiments, those components are usually delivered by AAV. In a diabetic rat model, AAV PHP.eB containing Nogo-B small interfering RNA was intravitreally injected, reducing the expression level of Nogo-B in the retina and further increasing the permeability of retinal blood vessels [116]. In another study, AAV2 expressing src homology 2 domain-containing protein tyrosine phosphatase-2 (SHP2) enhanced green fluorescent protein-short hairpin RNA was administered intravitreally in the eyes of mice, resulting in the knockdown of SHP2 gene, which plays a critical role in various intracellular pathways of multiple growth factor receptors [117]. Through IVT injection, AAV ShH10 is believed to be able to transduce mouse ciliary epithelial cells, which contain the CRISPR-Cas9 system that can disrupt the aquaporin 1 gene, leading to a reduction in intraocular pressure [118]. Recently, the CRISPR interference (CRISPRi) system was developed for repression of the neural retina leucine zipper (NRL) gene without causing double-strand breaks (DSBs) or genomic changes, which is considered to be much safer than CRISPR-mediated gene disruption. In this system, the dCas9 enzyme is divided into 2 parts, in which the N terminus and Kruppel-associated box (KRAB) reporter domain are fused and delivered by one AAV, and the C terminus and single guide RNA targeting the NRL gene exon are delivered by another AAV. Such a dual AAV delivery system can completely reconstruct the dCas9 enzyme, silencing the NRL gene with similar effects as the NRL knockout approach. The CRISPRi system successfully transformed the rods of RD10 mice into cone-like cells, prolonging photoreceptor survival and preserving visual behavior [119].

**Table 4.** Major gene silencing and editing strategies for treatment of ocular diseases

Strategy	DSB	Gene editing	Gene silencing	Donor template required	Limitation
CRISPR-Cas9	Yes	Yes	Yes	HDR (with template)/NHEJ (without template)	The efficiency of HDR in nondividing cells is relatively low.
CRISPR-Cas13	No	No	Yes	No	Off-target effect at RNA level. Off-target effect
Interfering RNA	No	No	Yes	No	Off-target effect
CRISPRi	No	No	Yes	No	Interference efficiency varies depending on the target gene and may affect the nearby genes
BE	No	Yes	No	No	Only suitable for single base editing (C to T or A to G), currently with low in vivo editing efficiency
PE	No	Yes		Yes (targeted insertions, deletions, and base-to-base conversions in both dividing cells and postmitotic cells)	At present, the ability to insert or delete large fragments of DNA has not been verified. Low in vivo editing efficiency

Although CRISPR technology is usually related to DNA editing, the emerging of the Cas13 enzyme, which cleaves RNA fragments through the intrinsic ribonuclease activity of CRISPR RNA guiding molecular binding activation, has promoted the creation of flexible and editable tools that can target RNA. Compared with Cas9, Cas13 family proteins are relatively small and easy to package into AAV. Compared to DNA editing, the advantages of RNA editing lie in its reversibility, as it rarely causes permanent changes to the genome. However, because of their collateral cleavage effect, the Cas13 family has serious off-target effects at the RNA level, which can also bring some potential risks. CasRx is a novel member of the Cas13d protein family. It has been proven at the Cas13d system targets silencing RNA in mice [120]. Furthermore, Sun et al. [121] reported that by AAV delivery, the newly developed RNA-targeted CRISPR system CasRx can knock down the expression of VEGFA mRNA, significantly reducing the size range of choroidal neovascularization and verifying the potential of RNA targeted CRISPR system in therapeutic applications. This study offered a safer choice for the future treatment of ocular dominant genetic diseases and provided a novel method for the management of many degenerative diseases.

## AAV-Mediated Gene Editing for Ocular Disorders

In previous clinical studies, gene therapy was mainly conducted by supplementing genes to produce functional proteins [122]. However, AAV-mediated gene replacement is not applicable to some ocular recessive diseases. Other aspects also limit the development of treatment using AAV platform, like gene replacement being insufficient for dominant mutations and the size of the therapeutic protein [7]. With the progress of gene editing technology, various types of gene editing agents are emerging to correct various types of genetic mutations precisely, making it possible for in vivo gene repair. Many inherited ocular degenerative diseases are induced by mutations in specific genes. For example, mutations in the RHO gene account for 8% to 10% of all RP cases, which is one of the most common causes of RP. There are more than 150 pathogenic mutations in RHO gene, most of which are point mutations [73]. However, because of technical limitations, not every RHO gene mutation site can be efficiently, safely, and accurately repaired. In 2018, Tsai et al. [115] reported a kind of strategy called “ablate-and-replace” to first destroy the endogenous alleles (WT and mutant RHO alleles) and followed with the exogenous WT RHO overexpression by gene supplementation. Since this strategy can treat diseases caused by multiple dominant mutations in the same gene and is suitable for different types of mutations, it is a relatively economical choice.

he first gene editing strategy based on CRISPR-Cas9 is to develop HDR pathways for promoting precise mutation correction with the assistance of exogenous DNA templates. The HDR pathway mainly occurs in the S and G2 phases of the cell cycle, and the repair efficiency is low for postmitotic retinal cells. Thus, current research focuses on improving the HDR efficiency. Postnatal rodless (rd1) mouse is an RP mutation model characterized by visual loss and photoreceptor degeneration. Cai et al. [123] invented a Cas9/RecA system based on HDR, which can accurately correct PDE6B mutations in rd1 mice and improve HDR efficiency.

Recently, new editing tools, base editors (BEs) and prime editors (PE), have been experimentally used for precise correction in postmitotic retinal cells without causing DSBs. The BE can accurately correct point mutations or single-nucleotide polymorphisms at the target position of genomic DNA. The BE system is mainly composed of deaminases and dead Cas9 (noncleaving activity) proteins. They will accurately target a sequence and convert one nucleotide to another. A split BE dual AAV system has been proven to be able to achieve base editing with therapeutic efficiency in mouse retina [124]. Furthermore, D. Liu’s team constructed a miniaturized and highly active ABE8e variant, saABE8e, and determined the least necessary cis-acting elements in the AAV genome to develop an efficient single AAV vector with extensive in vivo targeting capability, improve editing efficiency, reduce the required AAV dose, and thus reduce potential toxic side effects. For most ocular inherited diseases caused by point mutations, the BE can be predicted to be widely used. Anzalone et al. [125] reported a “search-and-replace” genome editing technique called PE, which consists of a prime editing guide RNA and reverse transcriptase fused to a Cas9 H840A nickase. PE is guided by the prime editing guide RNA to identify specific sequences on DNA. This tool can correct genomic changes not only point mutations but also short insertions or deletions without double-stranded cleavage [125]. Similar to the BE system, researchers have reported that in human cells, split PEs delivered by double AAV1 can mediate the insertion and base conversion of 4 endogenous sites [44]. PE tools can cover most known types of human pathogenic mutations. Although there are currently relatively few reports on the use of PE system in optical gene therapy, the PE system has bright prospects in ocular gene therapy on account of its wide selection of targeted sites, high editing efficiency, and low off-target rate [126,127].

On 25 July 2019, Editas launched a clinical trial of CRISPR-Cas9 in vivo gene editing EDIT-101, aimed at correcting genetic defects in retinal photoreceptors and treating LCA10. This is also the world’s first gene editing therapy to enter clinical practice. EDIT-101 is a drug based on CRISPR system, developed by Allergan and Editas Medicine, aiming at treating LCA10 caused by intron mutations in CEP290 gene (c.2991+1655A>G) [128]. Recently, Editas Medicine has released new data on its in vivo gene editing therapy EDIT-101 in a phase I/II clinical trial for treating LCA10. The data show that EDIT-101 exhibits good safety in all dose cohorts. This clinical trial recruited 14 patients, and only 3 patients achieved clinically marked visual improvement after EDIT-101 treatment. Moreover, 2 homozygous patients all had reactions, but only one of the 12 heterozygous patients had reactions. This also means that EDIT-101 has a poor therapeutic effect on heterozygous LCA10 patients, while it has a slightly better effect on homozygous LCA10 patients. However, because of the limited number of eligible subjects, the company has suspended recruitment for the clinical trial and will continue to conduct long-term follow-up for all patients who have received treatment. Although it is still limited by the number of patients and efficacy, EDIT-101 is a important progress in gene editing for the treatment of ocular disorders, and more gene editing methods will be attempted for the treatment of those disorders and promoted to clinical application.

## Evolution of AAV Capsid Engineering

The transduction efficiency and ocular tissue tropism vary among different AAV serotypes [129]. The genetic modification of AAV vectors can enhance their tissue tropism, transduction efficiency, and the ability of the transgene and capsid to escape from host immune response [9]. The capsid characteristics of AAV determine which kind of cells will be transduced once AAV is transported near the tissue. According to the management of different delivery routes, AAV with diverse capsids can reach various types of ocular cells. In general, AAV will transfect most of the cells in contact with it. In addition, AAV capsid interacts with corresponding cell surface receptors, which mediate the entry of AAV of different serotypes into cells and nuclei [28]. At present, there are 13 natural AAV serotypes, among which AAV2 is the most widely used in gene therapy clinical trials [48]. Many researchers, based on AAV2, have engineered its capsid to enhance its gene delivery efficiency in various ocular cells, looking for new AAV capsid variants for efficient delivery in the eye and trying to optimize AAV-mediated eye gene therapy [53]. Rational design is one of the earliest methods used for AAV capsid engineering. This strategy begins by simply transplanting peptide fragments that bind to cell-type-related receptors [130]. By inserting random nucleic acid sequences encoding peptide libraries, including using a rational design to generate a new AAV capsid, it can transfer genes to cells that are usually tough to be transduced and insert specific receptor binding peptides on target cells into allowed sites in the AAV capsid genome, like 587 or 588 residues of AAV2 VP1 [131,132]. Using an in vivo capsid evolution strategy, two novel AAV AAV2.GL and AAV2.NN were developed through capsid recombination. In vivo test of animal models (mice, dogs, and monkeys) shows that those 2 novel AAV variants can efficiently target retinal photoreceptors by simply injecting them into the vitreous of the eyes. These new AAV vectors circumvent the disadvantage that the SR injection method may cause damage to fragile retinal tissue, and the AAV virus has a weak diffusion ability after SR injection [133]. Directed evolution is an effective strategy for AAV variant selection. In AAV-directed evolution, through circulating screening of some capsid gene mutations in eye tissues, the capsid sequence with higher transduction efficiency is determined. AAV variants screened from rodent models do not necessarily play a role in primates. For example, 7m8 variants were screened by IVT injection. Vitreous injection in rodents can lead to highly extensive and efficient transduction in the whole layer of the retina. However, studies have found that the effect of 7m8 in primates is questionable since the limitation of thicker internal limiting membrane of primates [134]. Differences in eye structures between rodents, nonhuman primates, and humans have brought difficulties in screening ideal AAV variants. Although AAV capsid engineering has been developed in rodent and nonhuman primate models, they may not be used for gene therapy of patients in the short term. As most natural AAV capsids may be verified by the FDA in human beings, these facts may make it more difficult for researchers to risk testing new generations of AAV in humans.

## Optogenetics for Ocular Gene Therapy

Degenerative eye diseases caused by genetic mutations are one of the main focuses of ocular gene therapy. However, despite the remodeling, most inner retinal neurons, including RGCs, still retain their function even in the in the late stages of degenerative diseases. Such functional cells are the foundation of restorative therapies such as optogenetics [135,136]. The loss of photoreceptor cells is a common situation in most IRDs, and optogenetics endows any neuron with light responsiveness through the expression of opsin, which is an attractive functional compensation strategy [137]. When the cone loses its outer segment, it is possible to restore the retinal photosensitivity by ectopic expression of microbial opsin in the inner cells. This degraded cone loses its outer segment or interacting neurons and can be transduced by carriers like AAV to realize the expression of opsin, rendering them sensitive to light. This provides more opportunities for gene therapy to save vision in advanced diseases [138]. The study of optogenetics-mediated vision restoring was first conducted by researchers who complete the expression of microbial opsin channel rhodopsin-2 (ChR2) in the RGCs of mice and marmosets’ retina [139,140]. The initial optogenetics tool, ChR2, is a member of the light-gated ion channels. To optimize the photogenetic treatment of IRDs, more research focuses on the engineering modification of ChR2 and other microbial opsins. By accumulating a large amount of experimental data in vivo test, microbial opsin-based optogenetics therapy has been transformed into increasing number of clinical trials.

As one of the most significant delivery vectors in optogenetic therapy, AAV is widely used in these studies. Efficient and selective expression of microbial opsin in different retinal cells through AAV has become a more precise gene therapy strategy. By using AAV2.7m8 vector, Gauvain and colleagues expressed microbial opsin ChrimsonR in RGCs with more effectively and made a vision restoration in no-human primates [135]. Another study achieved enhanced expression of optogenetic transgene in bipolar cells by modifying the capsid and promoter of AAV, evoking high-frequency spiking responses in RGCs of previously blind, rd1, mice [141]. At present, none of these modified AAV vectors have been applied to optogenetic therapy for patients. We hope that since the better cell transduction efficiency of these new generation AAV vectors, they may convert more retinal cells into artificial photoreceptors, thereby improving the visual quality of patients.

## Conclusion

Considering its safety and therapeutic efficacy, the AAV vector is a promising gene delivery choice. Compared with other viruses, AAV has low immunogenicity, although the immunogenicity of AAV relies on many factors, for instance, the promoter, the mRNA of the transgene, the ITR structure on both sides of the vector, the capsid of AAV, and the dosage and the injection route [142]. AAV vectors have a lower host gene integration rate, so compared with other viral delivery vectors, AAV has fewer changes in the host genome sequence, and its clinical application is considered more reasonable [143]. AAV has been effectively applied in several animal tests for long-term gene expression and has been stably used in clinical practice for more than 10 years [47].

AAV is considered the safest gene therapy vector and has been widely studied and applied, but its safety issues still have some controversy, mainly focusing on 3 aspects: carcinogenicity, hepatotoxicity, and immunogenicity. Last year, Novartis reported that 2 children died of acute liver failure after receiving Zolgensma treatment. In fact, as early as 2018, relevant studies had shown that AAV carriers have high affinity for the liver and naturally accumulate in liver cells. This fact leads to the fact that if only targeting the liver, low-dose AAV carriers are effective, but if targeting other parts, to achieve effective concentration, the AAV carrier dose during systemic administration must be significantly increased, and considering the empty virions, the actual liver burden of patients is heavier. The first-generation AAV2 vector was used in the first gene therapy clinical trial targeting the liver for the treatment of hemophilia B. There were related risk reports indicating that when the AAV vector dose was increased in gene therapy, CD8^+^ T cells responded to the capsid protein, causing the AAV vector to be cleared by the human immune system before cell transduction [144]. Recently, researchers have found that some therapeutic transgene fragments delivered by AAV are integrated into the vicinity of growth control genes on dog chromosomes, which has the potential to induce cancer [145]. Fortunately, because of the relatively closed environment and local delivery methods of the eyes, the potential systemic delivery risks of AAV can be effectively avoided in gene therapy for eye diseases, making AAV one of the most promising delivery tools for ophthalmic diseases. However, the transgene and capsid protein are known foreign inflammation inducers. In the case of AAV-mediated retinal gene therapy, there was also a loss of efficacy after initial functional improvement and intraocular inflammation. Previous studies have demonstrated that AAV can activate innate pattern recognition receptors, like Toll-like receptors (TLR-9 and TLR-2), thereby promoting the release of inflammatory cytokines and type I interferons. AAV can also neutralize anti-AAV antibodies and induce transgenic and capsid-specific T cell responses, both of which limit the therapeutic effect [31]. In primates, both SR and IVT administration of AAV vectors induced mild dose-dependent inflammation. AAV vectors in the vitreous cavity more easily contact the immune system than those in the SR space [146,147]

The packing capacity of AAV is also another key factor limiting its application in ocular gene therapy. Although the dual AAV system is expanding its packing capacity, its therapeutic gene expression efficiency will also be affected. Recently, researchers used multiple AAV vectors to deliver DNA fragments through split introns, enabling trans-splicing of DNA fragments and achieving full-length ATP binding cassette subfamily A member 4 (ABCA4) gene (6.8 kb) in human retinal organoids and retinal cells of animal model, thus providing a kind of treatment strategy for Stargardt’s disease, the most common hereditary macular dystrophy in adolescents [148]. The synthesis of double-stranded DNA is the main step limiting the transduction rate of AAV, and the ITR in single-stranded AAV (ssAAV) is the main switch in this step. By mutating the ITR structure of WT AAV, the step of second-chain synthesis after transduction can be skipped. Compared to single-stranded AAV, the self-complementary AAV (scAAV) vector expressed faster and had an enhanced of transgenic expression. The major disadvantage of scAAV vectors is that compared to traditional AAV vectors, they have a smaller packing capacity of only 2.5 kb. Efficient, long-lasting, and safe expression of transgenes is the ultimate goal of AAV as a delivery tool.

Liu’s team [125,149,150] has developed novel gene editing tools, called BEs and PEs, which can complete accurate gene editing without introducing breaks into DNA. These technologies are relatively new. Within 5 years, these technologies have been evolving continuously, breaking through the restrictions and optimizing their use in mammals. AAV delivery gene editing tools make more choices for ocular gene therapy [151]. Research in this field is still in the mouse experimental stage, and some potential side effects may yet to be revealed. More nonhuman primate experiments need to be conducted to verify its effectiveness and safety and can finally go to the clinic. Another new idea of AAV-mediated gene therapy is not confined to WT gene complementation or correction of pathogenic gene. As we gain a deeper understanding of the pathogenesis of ocular diseases, AAV can also be applied to target the processes and pathways related to mutated genes, which has expanded the types of diseases it applies to, such as AMD, complex glaucoma, and other disorders [152].

The results from AAV-mediated ophthalmic RCT are still increasing and will continue to increase, concerning findings that were not clearly confirmed in animal test or in currently ongoing clinical studies. At present, although there are still many limitations, AAV vectors are valuable and potential clinical tools for ocular gene therapy. It can be a long and tough process for medication based on the AAV vector from “bench to bedside”. As the initial FDA-approved ophthalmic gene therapy drug, Luxturna makes patients regain their brightness by supplementing RPE65. AAV-mediated gene therapy is expected to overcome some diseases that cannot be solved by traditional ophthalmic diagnosis and treatment methods. For this reason, it has provided treatment methods for some ocular diseases previously considered incurable diseases and has provided increasingly better options for the treatment of many traditional disorders.
